# Dynamic atomic observations in electrochemical interfaces

**DOI:** 10.1093/nsr/nwae297

**Published:** 2024-08-24

**Authors:** Hao Zhang, Yu Zhang, Zhenhai Wen

**Affiliations:** Department of Chemical Engineering, Massachusetts Institute of Technology, USA; Department of Chemistry, University of Oxford, UK; CAS Key Laboratory of Design and Assembly of Functional Nanostructures, and Fujian Provincial Key Laboratory of Materials and Techniques toward Hydrogen Energy, Fujian Institute of Research on the Structure of Matter, Chinese Academy of Sciences, China; CAS Key Laboratory of Design and Assembly of Functional Nanostructures, and Fujian Provincial Key Laboratory of Materials and Techniques toward Hydrogen Energy, Fujian Institute of Research on the Structure of Matter, Chinese Academy of Sciences, China

Direct detection of the solid-liquid electrochemical interface (ESLI) dynamics at the atomic scale is crucial for understanding and optimizing electrochemical reactions [1], yet traditional imaging techniques struggle with capturing these details due to the masking effects of liquid environments and the complexity of dynamic processes [2]. In recent years, researchers have been able to achieve real-time observation and analysis of ESLI structural changes and behaviors during electrochemical reactions at high resolution, facilitated by advancements in *in-situ* liquid cell technology integrated with advanced imaging and theoretical computational methods, especially *in-situ* transmission electron microscopy (TEM) [3]. These developments are crucial for understanding the behavior of materials in batteries and other electrochemical systems at the atomic scale, which can lead to better performance and longevity.

**Figure 1.**
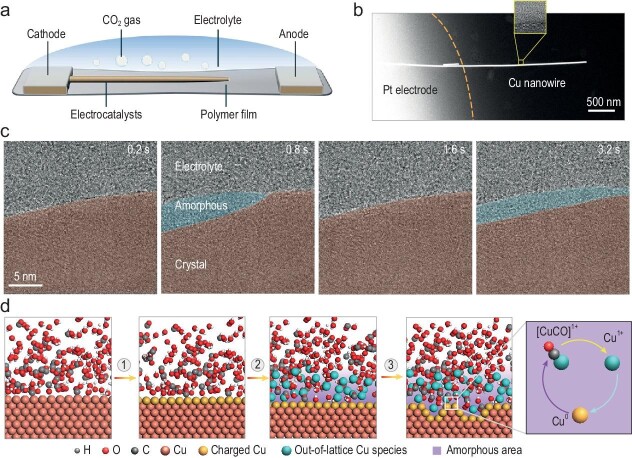
Experimental setup, characterization and proposed formation mechanism of the fluctuating liquid-like amorphous interphase. (a) A schematic view of the sample area. (b) HAADF image of a Cu nanowire suspended on the Pt electrode. The inset highlights the ESLI. (c) TEM images showing the emergence and fluctuations of the amorphous interphase. (d) Schematic illustration of the restructuring process of crystalline Cu surface, including activation of the surface atoms, dissolving of Cu and combination with solvent molecules, and reversible transformation between amorphous interphase and crystalline Cu. Adapted from Ref. [4].

Zheng's group, through the development of an advanced electrochemical liquid cell with polymer electrolyte (Fig. 1a), has for the first time directly observed ESLI atomic dynamics by using TEM (Fig. 1b and c), and proposes a mechanism of amorphous interphase formation [4]. Their study reveals a fluctuating liquid amorphous intermediate phase in the copper-catalyzed CO_2_ electroreduction reaction (CO_2_ER), which appears and disappears under electrochemical bias, influencing the restructuring and mass loss of crystalline copper surfaces. High-resolution imaging, along with EDS and EELS analyses, identifies the amorphous intermediate phase composed of copper, oxygen, and hydrogen, exhibiting dynamic behaviors including flow along the crystalline copper surface and mutual transformation with crystalline copper. Density functional theory (DFT) calculations elucidate the mechanism of copper atom removal during the electrochemical reaction (Fig. 1d). These findings provide crucial experimental and theoretical insights for further research and optimization of electrocatalysts.

The paper integrates various methods including TEM imaging, EDS, and EELS analyses to comprehensively characterize the composition and structure of the amorphous intermediate phase, ensuring the reliability and accuracy of the results. Compared to other recent works, such as studies focusing on the static observation of ESLI or those using less advanced imaging techniques, Zhang *et al.*’s approach offers a more detailed and dynamic understanding of the interface. The ability to monitor changes at the atomic level in real-time provides a significant advantage in tailoring materials for specific electrochemical applications, potentially leading to breakthroughs in catalyst design and battery technology. This research not only pushes the boundaries of what can be observed using *in-situ* TEM but also opens up new avenues for improving electrochemical systems by considering the previously overlooked dynamics of the amorphous interphase.

However, challenges remain in TEM-based observation of electrochemical reactions: the experimental setup and techniques are highly complex, requiring advanced equipment and operational skills, which limits widespread adoption and application; precise control of environmental conditions (e.g. temperature, pressure) remains challenging, potentially affecting the reproducibility and reliability of experimental results; the study primarily focuses on copper-catalyzed CO_2_ electroreduction, necessitating further validation of applicability across other electrochemical reactions and material systems [5].

Improvements in TEM-based observation methods for electrochemical reactions are suggested as follows: develop simpler and more user-friendly experimental setups to reduce dependency on advanced equipment and operational skills, thereby promoting wider applications of the method; enhance experimental setups to improve precision in environmental condition control, ensuring reproducibility and reliability of experimental results; expand research to include different electrochemical reactions and material systems to verify method applicability and explore various electrochemical interface phenomena; increase observation of long-term dynamic behaviors during electrochemical reactions to investigate time-evolution patterns and stability issues in depth; integrate macroscopic and microscopic scale analysis methods, such as *in-situ* electrochemical analysis techniques, to comprehensively understand the characteristics and mechanisms of electrochemical reactions.
